# Microbial Community Patterns Associated with Automated Teller Machine Keypads in New York City

**DOI:** 10.1128/mSphere.00226-16

**Published:** 2016-11-16

**Authors:** Holly M. Bik, Julia M. Maritz, Albert Luong, Hakdong Shin, Maria Gloria Dominguez-Bello, Jane M. Carlton

**Affiliations:** a Center for Genomics and Systems Biology, Department of Biology, New York University, New York, New York, USA; b Human Microbiome Program, New York University School of Medicine, New York, New York, USA; University of Iowa

**Keywords:** 16S rRNA, 18S rRNA, New York City, automated teller machine, environmental sequencing, urban microbiome

## Abstract

In densely populated urban environments, the distribution of microbes and the drivers of microbial community assemblages are not well understood. In sprawling metropolitan habitats, the “urban microbiome” may represent a mix of human-associated and environmental taxa. Here we carried out a baseline study of automated teller machine (ATM) keypads in New York City (NYC). Our goal was to describe the biodiversity and biogeography of both prokaryotic and eukaryotic microbes in an urban setting while assessing the potential source of microbial assemblages on ATM keypads. Microbial swab samples were collected from three boroughs (Manhattan, Queens, and Brooklyn) during June and July 2014, followed by generation of Illumina MiSeq datasets for bacterial (16S rRNA) and eukaryotic (18S rRNA) marker genes. Downstream analysis was carried out in the QIIME pipeline, in conjunction with neighborhood metadata (ethnicity, population, age groups) from the NYC Open Data portal. Neither the 16S nor 18S rRNA datasets showed any clustering patterns related to geography or neighborhood demographics. Bacterial assemblages on ATM keypads were dominated by taxonomic groups known to be associated with human skin communities (*Actinobacteria*, *Bacteroides*, *Firmicutes*, and *Proteobacteria*), although SourceTracker analysis was unable to identify the source habitat for the majority of taxa. Eukaryotic assemblages were dominated by fungal taxa as well as by a low-diversity protist community containing both free-living and potentially pathogenic taxa (*Toxoplasma*, *Trichomonas*). Our results suggest that ATM keypads amalgamate microbial assemblages from different sources, including the human microbiome, eukaryotic food species, and potentially novel extremophilic taxa adapted to air or surfaces in the built environment. DNA obtained from ATM keypads may thus provide a record of both human behavior and environmental sources of microbes.

**IMPORTANCE** Automated teller machine (ATM) keypads represent a specific and unexplored microhabitat for microbial communities. Although the number of built environment and urban microbial ecology studies has expanded greatly in recent years, the majority of research to date has focused on mass transit systems, city soils, and plumbing and ventilation systems in buildings. ATM surfaces, potentially retaining microbial signatures of human inhabitants, including both commensal taxa and pathogens, are interesting from both a biodiversity perspective and a public health perspective. By focusing on ATM keypads in different geographic areas of New York City with distinct population demographics, we aimed to characterize the diversity and distribution of both prokaryotic and eukaryotic microbes, thus making a unique contribution to the growing body of work focused on the “urban microbiome.” In New York City, the surface area of urban surfaces in Manhattan far exceeds the geographic area of the island itself. We have only just begun to describe the vast array of microbial taxa that are likely to be present across diverse types of urban habitats.

## Abstract (precis)

Automated teller machine (ATM) keypads represent a specific and unexplored microhabitat for microbial communities. Although the number of built environment and urban microbial ecology studies has expanded greatly in recent years, the majority of research to date has focused on mass transit systems, city soils, and plumbing and ventilation systems in buildings. ATM surfaces, potentially retaining microbial signatures of human inhabitants, including both commensal taxa and pathogens, are interesting from both a biodiversity perspective and a public health perspective. By focusing on ATM keypads in different geographic areas of New York City with distinct population demographics, we aimed to characterize the diversity and distribution of both prokaryotic and eukaryotic microbes, thus making a unique contribution to the growing body of work focused on the “urban microbiome.” In New York City, the surface area of urban surfaces in Manhattan far exceeds the geographic area of the island itself. We have only just begun to describe the vast array of microbial taxa that are likely to be present across diverse types of urban habitats.

## INTRODUCTION

In recent years, the growing accessibility of high-throughput sequencing technologies has vastly expanded our knowledge of microbial communities on a global scale, encompassing both natural and human-made ecosystems. Environmental sequencing studies focusing on conserved, phylogenetically informative genetic loci (e.g., nuclear markers encoding ribosomal subunits, such as the 16S rRNA gene in bacteria/archaea and the 18S rRNA gene in eukaryotes [1]) have enabled rapid detection and description of uncultivated taxa across diverse ecosystems. The majority of studies have sought to describe microbial biodiversity and assess ecological patterns of “pristine” natural habitats such as oceans (2), lakes (3), soils (4, 5), sea ice and glaciers (6), and even clouds (7). More recently, however, a number of studies have aimed at directly assessing human contributions and their impacts on microbial communities. Studies of the built environment have sought to understand how building architecture and engineering, in conjunction with human behavior, may influence the microbes that we encounter during our time spent indoors (which represents 87% of our time on average [8]). Such studies have aimed at capturing the microbial communities associated with both air and surfaces in homes (9, 10), hospitals (11, 12), classrooms (13), offices (14, 15), and restrooms (16), as well as community assemblages within plumbing systems (17–19) and an expanding set of other indoor microhabitats.

In a similar but distinct vein, studies of urban ecosystems have aimed to understand the processes and factors governing microbial communities in metropolitan areas worldwide, where the density of humans is great but the ecosystems themselves are more sprawling and open to the elements. In urban ecosystems, the distribution and diversity of microbes may thus be shaped by a combination of both ecological and human-mediated processes. To date, urban environmental sequencing work has sought to describe microbial communities associated with urban air (20–24), rodents (25), and urban soils in green roofs, city parks, and road medians (26, 27), as well as surfaces within urban transit systems (28, 29). However, those studies captured only a small part of the microbial diversity that is likely to be present in urban environments, and there are many other types of locations, substrates, and surfaces which may serve to deepen our knowledge of microbial ecology and public health in metropolitan areas.

**FIG 1:**
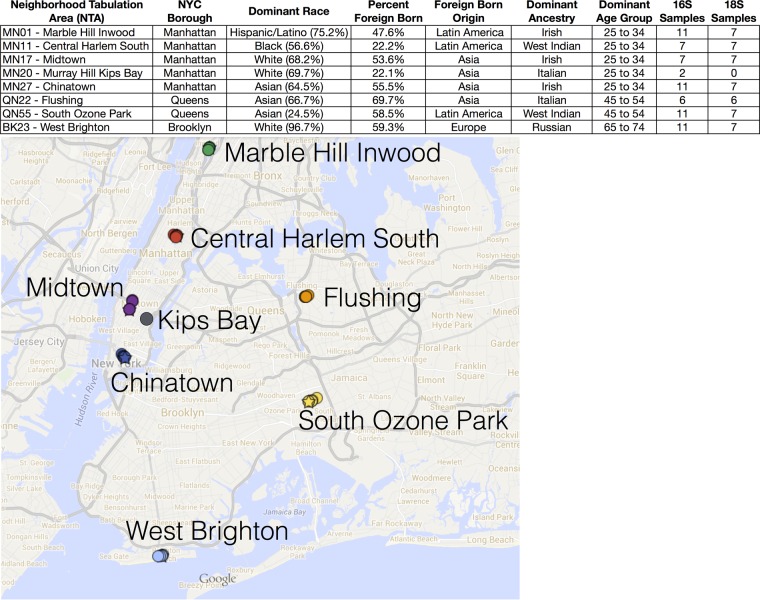
Map and population demographic metadata of sample sites in New York City. Microbial swab samples were collected at automated teller machines (ATMs) in eight neighborhood tabulation areas (NTAs), representing three boroughs of New York City (Manhattan, Queens, and Brooklyn). NTA population demographics, representing 5-year estimates from the United States Census Bureau’s American Community Survey (ACS) (2008 to 2012), were obtained from the NYC open data portal (https://nycopendata.socrata.com/). “ancestry” demographics represent write-in responses from a small subset of survey respondents, enabling respondents to report ethnic origins that are not otherwise captured in questions pertaining to race or foreign-born status in the ACS. Age data represent years. (Map data © 2016 Google.)

To characterize microbial diversity and biogeography in a unique (but insufficiently studied) component of the urban landscape, we carried out a baseline survey of microbial community diversity associated with automated teller machine (ATM) keypads in New York City (NYC). The geography, population density, and accessibility of demographic data in NYC provide a unique case study—in terms of floor space, the “indoor biome” of Manhattan is three times as large as the geographic area of the island itself (30). With this focus on NYC, we aim to facilitate the assessment of different factors that may govern microbial biodiversity and community structure in specific urban habitats. The ATM keypad can be considered a highly trafficked surface that routinely comes into contact with human inhabitants, similarly to railings, seats, and turnstiles in urban transit systems (28, 29). Several culture-based studies of ATMs around the world have previously been carried out (31–33), but no study to date has yet applied high-throughput sequencing methods to deeply characterize all taxa which may be present on ATM keypads. In the present study, we sampled ATM keypads across eight neighborhoods in three NYC boroughs, where US Census data indicated the presence of demographically distinct populations of local residents (Fig. 1). Our goal was to broadly characterize the microbial assemblages recovered from ATM keypads, using an environmental sequencing workflow that concurrently recovered bacterial/archaeal taxa (16S rRNA gene amplicons) as well as microbial eukaryote communities (18S rRNA gene amplicons). The parallel collection of sample metadata and neighborhood census data also allowed us to assess whether microbial biogeography in NYC was correlated with ATM characteristics, local population demographics, or geographic factors. Statistical analyses were further implemented to determine the potential source of ATM microbial assemblages (e.g., the human microbiome, air, food, etc.) as well as potential biomarkers associated with different sample classes.

## RESULTS

### Influence of OTUs from control samples.

We collected 66 samples from ATM keypads across New York City (8 neighborhoods in Manhattan, Queens, and Brooklyn; Fig. 1), including six control swabs that were exposed to ambient air at different sites. The sampling strategy was designed to target geographic areas with distinct ethnic and population demographics, known as neighborhood tabulation areas (NTAs), defined by the NYC Department of City Planning (see Materials and Methods). The majority of ATMs were sampled from indoor locations (62 samples were taken inside buildings or vestibules); however, a small subset of ATM keypads represented outdoor sample locations (4 samples [included in the 16S rRNA sequencing run only]). During initial analysis of 16S and 18S rRNA datasets, control samples formed a distinct grouping separate from the ATM samples in Unifrac principal-coordinate analyses (PCoAs) (see Fig. S1 in the supplemental material). Upon further investigation, our initial SourceTracker analysis revealed that a significant proportion of ATM sequences represented microbial operational taxonomic units (OTUs) that were also present in ambient air controls (Fig. S2 and S3). Examination of OTU tables suggested that the microbial OTUs present in control samples represent a mix of aerial microbes (e.g., fungal species and bacteria attached to dust particles, pollen, etc.), microbes present in the cotton swabs when purchased from the manufacturer, and “kit microbiomes” consisting of microbes derived from laboratory reagents (we recovered many known kit-associated bacterial genera such as *Acinetobacter*, *Pseudomonas*, *Deinococcus*, *Sphingobium*, and *Corynebacterium* [34]) or potential contamination introduced at some point during PCR and sequencing protocols. The OTUs sequenced from blank control samples most likely represent microbes from a combination of these sources; after assessing control samples and conducting SourceTracker analysis, we adhered to stringent data filtering protocols and subtracted all control sample OTUs from the entire data set.

10.1128/mSphere.00226-16.2 Figure S1  Unweighted Unifrac PCoAs showing distinct clustering of control samples. PCoAs were performed using abundance-filtered OTU tables, after removal of chimeras and OTUs that failed to align to reference rRNA databases. (A) 16S rRNA data for bacterial/archaeal taxa rarefied at 2,200 sequences per sample. (B) 18S rRNA data for eukaryotic taxa rarefied at 25,000 sequences per sample. Download Figure S1, PDF file, 0.1 MB. Copyright © 2016 Bik et al. 2016 Bik et al. This content is distributed under the terms of the Creative Commons Attribution 4.0 International license.

10.1128/mSphere.00226-16.3 Figure S2  SourceTracker results for 16S rRNA data before subtracting OTUs from control samples. SourceTracker analyses were performed on closed-reference OTUs from this study and 12 public “source” datasets obtained from a previous meta-analysis (68). All “kit control” data represent sequence reads generated from control swab samples in the present study. Download Figure S2, PDF file, 0.2 MB. Copyright © 2016 Bik et al. 2016 Bik et al. This content is distributed under the terms of the Creative Commons Attribution 4.0 International license.

10.1128/mSphere.00226-16.4 Figure S3  SourceTracker results for 18S rRNA data before subtracting OTUs from control samples. SourceTracker analyses were performed on open-reference OTUs obtained from the present study only (with control samples marked as a potential “source”). All “kit control” data represent sequence reads generated from control swab samples in the present study. Download Figure S3, PDF file, 0.1 MB. Copyright © 2016 Bik et al. 2016 Bik et al. This content is distributed under the terms of the Creative Commons Attribution 4.0 International license.

### Alpha and beta diversity analyses.

Assessment of alpha diversity suggested that the phylogenetic diversity of microbial communities on ATM keypads had been adequately captured by the sequencing workflows in this study (Fig. S4). Rarefaction curves calculated from stringently filtered OTU tables (subjected to abundance-based OTU filtering and subtraction of all control OTUs) were observed to be almost flat (for eukaryotic 18S rRNA data rarefied at 8,900 sequences per sample; Fig. S4B) or to be beginning to level off (for 16S rRNA data rarefied at 1,700 sequences per sample; Fig. S4A) within each of the eight NYC neighborhoods sampled. The differences in the shapes of the rarefaction curves were most likely due to the increased sequencing effort per sample for 18S rRNA amplicons and to the putatively lower phylogenetic diversity of microbial eukaryote taxa in urban environments.

10.1128/mSphere.00226-16.5 Figure S4  Alpha diversity by neighborhood calculated using Faith’s phylogenetic diversity metric. Rarefaction curves were calculated in QIIME using abundance-filtered OTU tables with control OTUs subtracted. (A) 16S rRNA data for bacterial/archaeal taxa rarefied at 1,700 sequences per sample. (B) 18S rRNA data for eukaryotic taxa rarefied at 8,900 sequences per sample. Download Figure S4, PDF file, 0.4 MB. Copyright © 2016 Bik et al. 2016 Bik et al. This content is distributed under the terms of the Creative Commons Attribution 4.0 International license.

**FIG 2:**
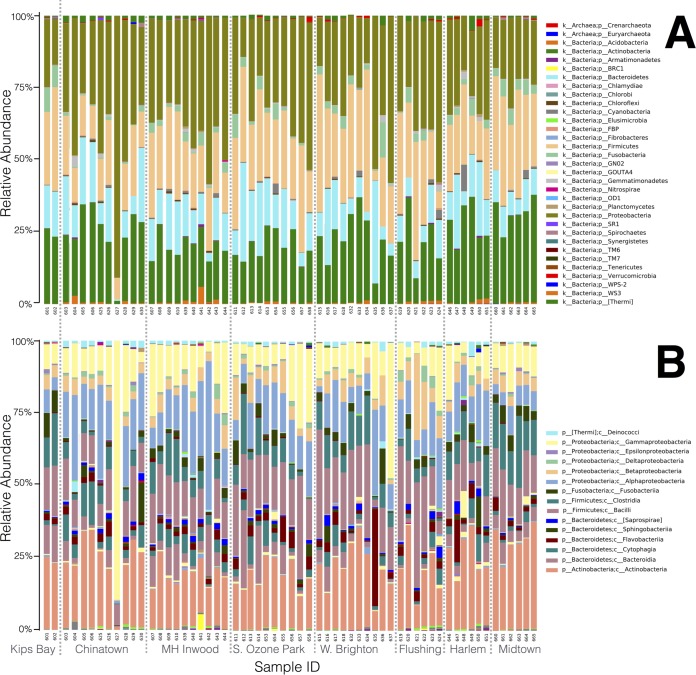
Relative abundances of bacterial/archaeal groups in 16S rRNA data set. (A) Microbial taxonomy summarized at phylum level. (B) Microbial taxonomy summarized at the class level; the legend displays only the top 15 most abundant taxa in the bar chart. Plots were generated in QIIME using abundance-filtered OTU tables with control OTUs subtracted. MH, Marble Hill; S., South; W., West.

**FIG 3:**
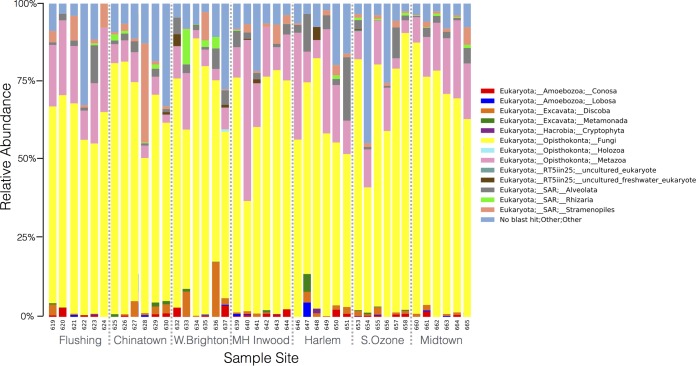
Relative abundances of eukaryotic groups in 18S rRNA data set. Summary of level 3 taxonomy data from the SILVA database, showing higher-level eukaryotic ranks observed in the ATM keypad data set. The plot was generated in QIIME using abundance-filtered OTU tables with control OTUs subtracted.

Taxonomy summaries for 16S and 18S rRNA showed that the major taxa recovered from ATM samples were largely consistent across datasets (Fig. 2 and 3). However, the presence or absence and relative abundances of other minor taxonomic groups were much more variable across samples. In the 16S rRNA data set, the most abundant bacterial phyla across most samples were *Actinobacteria*, *Bacteroides*, *Firmicutes*, and *Proteobacteria* (Fig. 2A); these taxa are representative of human skin communities and have been previously shown to dominate urban surfaces in the Boston subway system (28). At the class level, *Actinobacteria*, *Bacilli*, *Clostridia*, *Alphaproteobacteria*, and *Gammaproteobacteria* showed the highest relative abundances across most samples (Fig. 2B). The *Alphaproteobacteria* are considered a widespread and metabolically diverse group of environmental bacteria and have also been shown to be associated with urban transit system surfaces (28). Only nine archaeal OTUs were observed at low relative abundances and restricted to a few samples in the final abundance-filtered OTU tables. Further work is needed to determine whether *Archaea* can truly be considered “rare taxa” on ATM keypads or, alternatively, whether the 16S rRNA primer set used in this study prevented recovery of the majority of archaeal taxa; alternative primer sets or shotgun metagenomic sequencing is needed to provide further insight. In the 18S rRNA data set, fungal OTUs represented the largest taxonomic proportion in most samples, with metazoa and unassigned OTUs (those with no BLAST hit) representing two other taxonomic categories with high relative abundances across most samples (Fig. 3). Protist lineages (*Amoebozoa*, *Alveolata*, *Rhizaria*) had low diversity, were present at much lower relative abundances, and showed more variability across samples. The majority of protist sequences were derived from *Alveolata* and represent free-living ciliates, particularly *Oligohymenophorea* and *Colpodea*, which are taxa commonly found in freshwater and soil habitats (35, 36). Three samples contained a >1% relative abundance of *Entamoeba* species (samples 632, 637, and 661 from ATM keypads in West Brighton and midtown), a genus of protists classed within the phylum *Amoebozoa* and generally associated with the intestinal tract (37). One sample contained a >10% relative abundance of *Silicofilosea* protists (sample 633 from West Brighton), an amoebal member of the *Rhizaria* group known to be associated with bacterivory and fungivory in soil ecosystems (38). Free-living trichomonads (*Monotrichomonas carabina* and *Ditrichomonas honigbergii* [39, 40]), as well as a gut-associated commensal (*Pentatrichomonas hominis*) typically found in humans and other mammals (41) and a species closely related to the human parasite *Trichomonas vaginalis* that was originally isolated from avian sources (*Trichomonas* sp. strain 5 AP-2012; GenBank accession no. JX512960), were also recovered from ATM keypads. Recent studies suggest that both of these host-associated trichomonads may exhibit zoonotic characteristics (i.e., transmission between humans, domesticated animals, and wildlife) (42). *Toxoplasma*, another zoonotic protist taxon (43), was also detected at >3% relative abundance on one ATM keypad (sample 632 from West Brighton).

**FIG 4:**
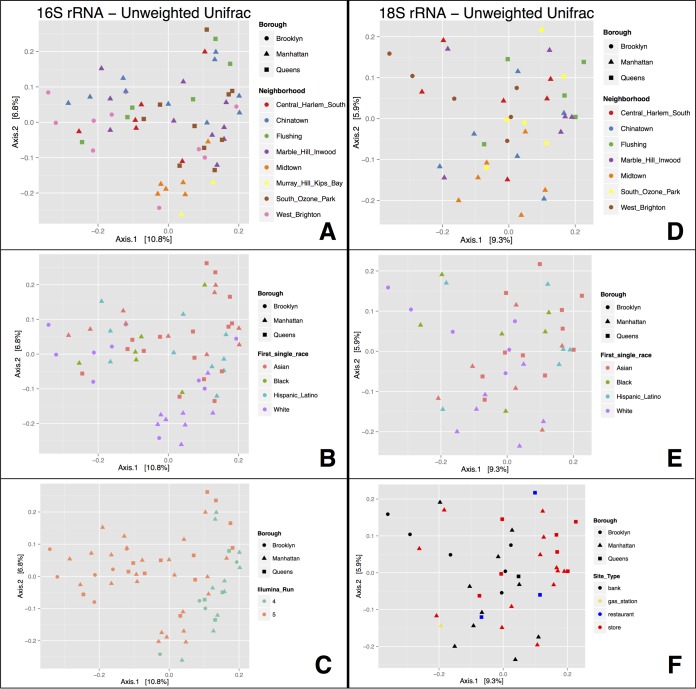
Beta-diversity analyses of microbial taxa recovered from ATM keypads. Data represent results of unweighted Unifrac PCoAs for 16S rRNA for bacteria/archaea (A to C) and 18S rRNA for eukaryotes (D to F), showing no obvious clustering of microbial assemblages according to NYC neighborhood (A and D), census population demographics (race group with highest proportion in each neighborhood) (B and E), or type of site where ATM was located (F). The strongest clustering pattern in the data set was a technical artifact observed for 16S rRNA samples sequenced across two Illumina MiSeq runs (C).

Beta diversity analyses of microbial communities revealed a lack of clear patterns across ATM keypads in New York City, and this absence of any obvious groupings was consistent across both prokaryotic (16S rRNA) and eukaryotic (18S rRNA) datasets (Fig. 4). In weighted and unweighted principal-coordinate analysis (PCoA) using Unifrac distances in QIIME, ATM samples showed no obvious clustering according to geography (neighborhood or borough; Fig. 4A and D), type of site where an ATM was located (bank, store, gas station, etc.; Fig. 4F), or local population demographic metadata obtained from online sources (predominant race group, age group, etc., in each NTA; Fig. 4B and E). Other factors such as date and time of sampling and material of ATM keypad (metal or plastic) also did not reveal any clear clustering of microbial communities (data not shown). Four outdoor ATMs were included in our sample set but were not included in 18S rRNA sequencing; while these four ATMs clustered together in 16S rRNA PCoAs, the corresponding outdoor samples were obtained from the same neighborhood (Chinatown, Manhattan). Small groups of samples from other neighborhoods were also observed to cluster together in our 16S rRNA data set (Fig. 4A), making it impossible to separate the influences of neighborhood and indoor/outdoor ATM location.

Datasets were assessed using various bioinformatic filtering strategies (abundance-based OTU filtering, differing levels of rarefaction), including approaches that both included (Fig. S1) and subtracted (Fig. 4) the microbial OTUs present in blank control samples. None of these methods produced strong groupings in PCoAs, suggesting that the lack of sample clustering across NYC ATMs represents a biologically valid result. However, permutational multivariate analysis of variance (PERMANOVA) tests revealed that the majority of sample groupings in unweighted Unifrac PCoAs are nonetheless statistically significant (Table 1). Borough and neighborhood were found to be statistically significant for both 16S and 18S rRNA datasets; additionally, ATM location (indoor/outdoor), population demographics (race), and Illumina run were statistically significant only in the 16S rRNA data set, and site type was statistically significant only in the 18S rRNA data set. These PERMANOVA results suggest that there may be some subtle differences in microbial community fingerprints across sample groups (e.g., that are revealed only by comparisons of Unifrac phylogenetic distances, as in this statistical test) that are not otherwise apparent in broader community comparisons such as those performed by PCoAs (Fig. 2).

**TABLE 1:** PERMANOVA test for statistical significance of sample groupings^a^

Category	16S rRNA (Bacteria/archaea)		18S rRNA (eukaryotes)	
	Pseudo-F value	*P* value	Pseudo-F value	*P* value
Borough	1.2744	**0.0279**	1.3959	**0.0028**
Neighborhood	1.3184	**0.0003**	1.1880	**0.0043**
Site type (Bank, store, etc.)	1.0497	0.2593	1.1942	**0.0257**
ATM location (indoor/outdoor)	1.7337	**0.0048**	NA	NA
Population demographics (race)	1.3559	**0.0038**	1.1141	0.1040
Illumina run	3.2439	**0.0001**	NA	NA

a Statistical tests were performed on unweighted Unifrac distance matrices (where PCoAs were generated from abundance-filtered OTU tables with control OTUs subtracted), using 10,000 permutations per test. Bold numbers represent significant *P* values of <0.05. Pseudo-F numbers represent F values by permutation.

During analysis, the strongest clustering pattern observed in our data set was a putative technical artifact resulting from 16S rRNA samples being split across two Illumina MiSeq runs (Fig. 4C). Data filtering and rarefaction did not effectively reduce or eliminate this technical artifact, and the Illumina run was found to be a strongly statistically significant sample grouping in PERMANOVA tests of Unifrac distances (*P* = 0.0001; Table 1). The persistence of such a technical artifact has been similarly reported in other recent studies (14). However, in our case, the 18S rRNA amplicons from eukaryotic communities were sequenced on a single Illumina run and thus provided an independent assessment of PCoA patterns. Eukaryotic PCoAs did not show any large differences in the microbial patterns by site (Fig. 4D and F).

### Source of microbial communities on ATM keypads.

**FIG 5:**
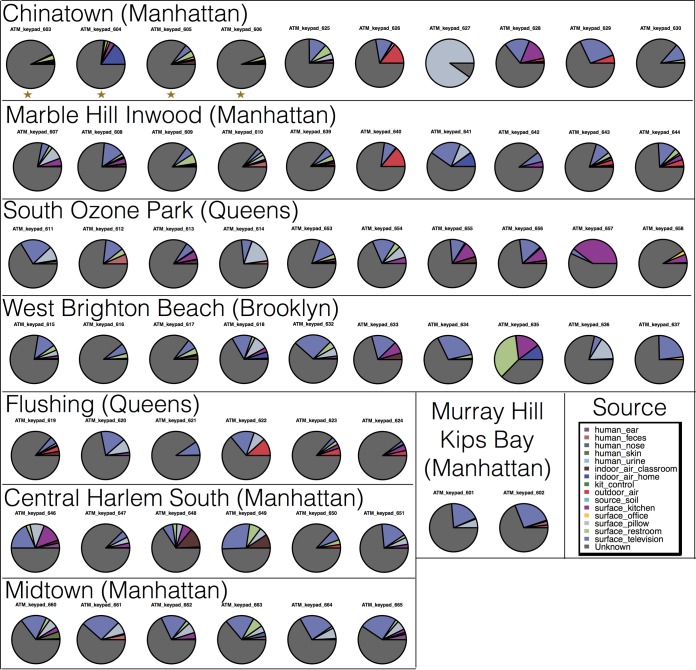
SourceTracker analysis of bacterial/archaeal assemblages on ATM keypads. Closed-reference OTUs (16S rRNA only) from this study were compared to 12 published datasets representing a range of potential source habitats (human body, building surfaces, indoor/outdoor air). The majority of microbes on each ATM keypad were derived from an unknown source. The most common identified source across all ATMs appeared to be household surfaces (rest room, kitchen, pillows, and televisions) and outdoor air. Gold stars denote the four ATMs in this study located at outdoor sites.

SourceTracker analysis carried out on the 16S rRNA data set indicated that the majority of microbes on each ATM keypad were derived from an unknown source (Fig. 5). For the majority of ATM samples, <25% of the microbial community was assigned to an identified source, although four samples (samples 627, 635, 646, and 649) were shown to have >50% of microbes assigned to known sources. Although this study included only four ATMs located outdoors (gold stars in Fig. 5), ATM location did not seem to influence the proportion of “unknown” sources for microbial OTUs, which was high across most samples. The most common identified sources of microbes on ATM keypads appeared to be household surfaces such as televisions, restrooms, kitchens, and pillows. In our SourceTracker analysis, we included data previously obtained from human hands and palms (44) as well as from other body sites such as the nose, ear, and gut (45). However, human skin and other body sites were not identified as dominant sources of ATM microbes in our SourceTracker analysis, despite the inclusion of 46 samples from two studies representing human skin (44, 45).

### Microbial biomarkers from LEfSe analysis.

**FIG 6:**
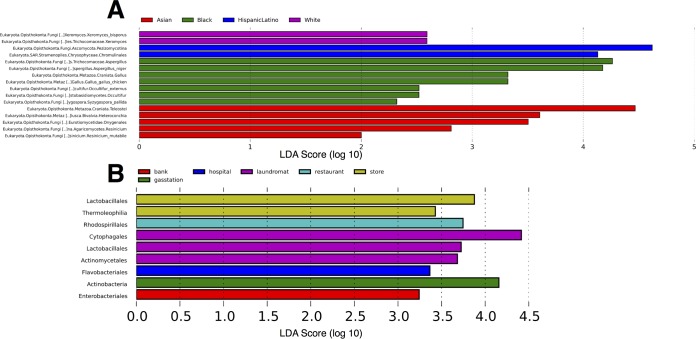
Linear discriminant analysis (LDA) effect size (LEfSe) analysis to determine microbial biomarker taxa across sample groups. (A) Eukaryotic 18S rRNA OTUs significantly enriched across census population demographics (predominant race group in each NTA). (B) Bacterial/archaeal genera significantly enriched across different ATM site types in 16S rRNA data set.

Linear discriminant analysis (LDA) effect size (LEfSe) analysis suggested the presence of a number of significant microbial biomarker taxa across different sample groupings (Table 2). In 16S rRNA datasets, geographic location (borough/neighborhood) represented the only sample grouping exhibiting no significant enrichment or depletion of microbial taxa. However, this pattern was not consistent for eukaryotes, where 7 to 36 biomarker taxa were attributed to geographic location in the 18S rRNA data set. Among all sample groups, the highest numbers of biomarker taxa were reported for “location” of ATMs within the 16S rRNA data set (keypads located indoors versus outdoors; Fig. S5); however, the type of site (bank, restaurant, gas station, etc.) and the population demographics associated with each NTA (predominant race group) were also associated with a low number of biomarker taxa in both 18S and 16S rRNA data (Fig. 6). Fungi comprised the majority of eukaryotic biomarkers identified in the 18S rRNA data set (Fig. 6A) and included both common species and specialized taxa. For example, the fungal species *Aspergillus niger* and *Occultifur externus* were both found to be enriched on ATM keypads sampled in Central Harlem South. *Aspergillus* species are ubiquitous and widespread fungal species associated with outdoor and indoor air (46, 47), and *A. niger* is a species heavily utilized in industrial processes (48) and the cause of black mold disease in many fruit and vegetable crops (49). In contrast, *O. externus* is a recently described novel species originally isolated from plant litter in Portugal (50). Furthermore, the xerophilic foodborne mold *Xeromyces bisporus* was reported as another fungal biomarker in NYC neighborhoods with predominately white population demographics (Fig. 6A); this fungal species has been reported to grow at extremely low water activity levels that are lower than those seen with any other known organism (51).

**TABLE 2:** Number of significantly discriminative taxa reported in LefSe analysis (absolute LDA score, >2.0)^a^

Category	No. of significantly discriminative taxa					
	16S rRNA (Bacteria/archaea)			18S rRNA (eukaryotes)		
	L5 taxa (family)	L6 taxa (genus)	OTUs	L5 taxa (family)	L6 taxa (genus)	OTUs
Borough/neighborhood	0	0	0	7	11	36
Site type (Bank, store, etc.)	2	9	3	3	5	25
ATM location (indoor/outdoor)	93	235	148	NA	NA	NA
Population demographics (race)	1	2	1	3	6	16

a LefSe analyses were performed on normalized BIOM tables from open reference OTU picking, following abundance-based filtering and removal of OTUs present in kit control samples. LefSe analyses were performed on OTU tables summarized at the L5 (family) and L6 (genus) taxonomy levels, as well as on unsummarized OTU tables. NA, ATM location comparisons were not possible for 18S rRNA, as only indoor ATMs were included in the eukaryotic data set.

10.1128/mSphere.00226-16.6 Figure S5  Linear discriminant analysis (LDA) effect size (LEfSe) analysis showing prokaryotic biomarker taxa across indoor/outdoor ATM keypads. Analysis was carried out on a 16S rRNA data set (normalized per sample) subjected to abundance-based filtering and subtraction of control sample OTUs. Download Figure S5, PDF file, 1.7 MB. Copyright © 2016 Bik et al. 2016 Bik et al. This content is distributed under the terms of the Creative Commons Attribution 4.0 International license.

A number of eukaryotic metazoan taxa appeared to be associated with population demographics in different NYC neighborhoods. Notably, bony fish (*Teleostei*) and molluscs (*Bivalvia*) were significantly enriched in ATM samples obtained from predominantly Asian neighborhoods (Flushing/Chinatown), while chickens (*Gallus gallus*) were significantly enriched in ATM samples obtained from a predominantly black neighborhood (Central Harlem South). In the 16S rRNA data set, ATM keypads located in laundromats and stores exhibited the highest number of biomarker taxa, with *Lactobacillales* significantly enriched across both site types (Fig. 6B). Overall, the number of significantly discriminative taxa was very low in LEfSe results from the 16S rRNA data set, with oftentimes only one enriched taxon reported per metadata class (Table 2).

## DISCUSSION

Here we present the first broad assessment of microbial communities associated with ATM keypads in New York City, characterizing assemblages of bacteria/archaea (66 samples) and microbial eukaryotes (48 samples) from eight NYC neighborhoods across Brooklyn, Queens, and Manhattan. This data set represents an important addition to the growing body of research focused on urban microbial ecology and specifically complements work performed in New York City which to date has focused on green roofs and park/median soils (26, 27), sewage (J. M. Maritz, K. H. Rogers, T. M. Rock, N. Liu, S. Joseph, K. M. Land, and J. M. Carlton, submitted for publication), rodents (25), and both air and surfaces within the NYC subway system (23, 24, 29). The results are of particular relevance with respect to humans, since the surfaces studied are touched by people and could potentially mediate interpersonal transfer of microbes or microbial DNA.

Unifrac principal-coordinate analysis indicated an overall lack of biogeographic patterns structuring microbial communities on ATM keypads in NYC (Fig. 4). This lack of any obvious pattern was consistent across both bacteria/archaea (16S rRNA) and microbial eukaryote (18S rRNA) datasets, suggesting that microbial community structure is not governed by any of the broad metadata categories (e.g., geography, population demographics, site type, data/time of sample collection, etc.) that we assessed during this study. The absence of biogeographic patterns could be explained by a number of factors. ATMs are subject to high use in urban areas such as NYC and could be subject to human-driven homogenization of the microbial communities present on keypads. In any given neighborhood, transient users of ATMs (tourists, commuters, visitors from other NTAs, etc.) might be common and might reduce/eliminate any specific microbial community signatures which might be associated with the population demographics in a given NTA (e.g., those related to age group, ethnicity, etc.). Furthermore, periodic cleaning or disinfection of ATMs, if implemented, may severely reduce the microbial diversity and prevent unique assemblages from accumulating or differentiating on ATM keypads across space and time.

Despite the lack of distinct clusters in Unifrac PCoAs (Fig. 4), LEfSe analyses identified a number of microbial biomarkers indicative of certain metadata classes in both 16S and 18S rRNA datasets. Across all metadata categories in the 18S rRNA data set, LEfSe analyses reported a large number of fungal biomarker taxa (e.g., Fig. 6). This suggests that localized enrichment of some fungal taxa—for example, enrichment of specific taxa that might represent a small fraction of the microbial community and thus might not represent strong enough enrichment to allow differentiation of overall microbial communities on Unifrac PCoAs—may be driving subtle biogeographic patterns in microbial eukaryote communities on ATM keypads. Previous studies have reported geographic partitioning and localization of urban fungal assemblages in NYC soils (27). It is unclear whether the fungal biomarkers on ATM keypads represent truly localized fungal diversity, or alternatively, stochastic enrichment of human-transported or airborne taxa.

In the eukaryotic data set, the most striking biomarkers appear to indicate a “molecular echo” (52) of food species on ATM keypads in certain neighborhoods (the domestic chicken *Gallus gallus* in Central Harlem South and bony fish [*Teleostei*] and mollusk [*Bivalvia*] species in Chinatown/Flushing; Fig. 6), potentially reflecting the concentrations of specialized restaurants in different areas of NYC. These food species appeared in the LEfSe results as significantly enriched OTUs within each respective neighborhood. While our study design does not allow us to pinpoint the exact source of such DNA, one reasonable explanation is that residual DNA from a recent local meal may persist on a person’s hands and be transferred to the ATM keypad upon use.

In addition to obvious species, another potential food biomarker is the fungal species *X. bisporus*, which was significantly enriched in midtown and other NYC neighborhoods with predominantly white population demographics. *X. bisporus* is a foodborne mold originally isolated from licorice and associated with spoilage of high-sugar foods such as cakes and confectionaries (51). Although we cannot confirm the original source of *X. bisporus* OTUs on ATM keypads, it seems plausible that this fungus had been transferred from people who had recently handled baked goods, particularly in a commuter-heavy area such as midtown Manhattan, where there are many nearby convenience stores and cafés selling this type of food product to business workers. A previous metagenomic analysis of the NYC subway system also detected food signatures across surfaces in the urban transit system (29), suggesting that genomic material from meals is routinely transferred around NYC by human inhabitants and may thus represent a common component of the urban microbiome.

The human microbiome represents an obvious source of microbial communities on ATM keypads; however, SourceTracker analysis did not pinpoint human skin or any other body site as a primary source of microbes (Fig. 5). In contrast, outdoor air and household surfaces—kitchens, restrooms, pillows, and televisions—were the most commonly identified source habitats. Household surfaces may effectively collect microbial communities from various sources (food, family members, pets, dust) and thus represent a pool of microbes originating from different habitats. This is in contrast to swab samples collected directly from human body sites (see, e.g., the published datasets [44, 45] used as human sources in this study), which represent the personalized microbiome associated with a single person. Since each ATM keypad in New York City is most likely utilized by hundreds of people each day (and may come into contact with air, water, and microbes from different urban surfaces), the microbial communities obtained in this study may represent an “average” community that is effectively pooled from vastly different sources (14). An alternative and potentially more plausible hypothesis is that the SourceTracker algorithm may be highly sensitive to the primer region and sequencing platform used to generate the “source” training sequences. The samples from the household surfaces representing the majority of assigned sources for ATM keypads were generated using the same primer set and sequencing technology utilized in this study (Illumina HiSeq/MiSeq data using the 515F/806R primers to amplify the 16S rRNA gene [10, 53]), which may explain why these household surfaces were identified as source habitats. In contrast, human microbiome “source” samples were generated using different primer sets and sequencing platforms (e.g., Roche 454), and the distinct rRNA region and lower-throughput nature of the sequencing technology may confound the ability of SourceTracker to assess source/sink habitats between these samples. Overall, the vast majority of ATM microbial communities were derived from an “unknown” source, and it is also possible that this unassigned community fraction represents human-associated microbes that SourceTracker was unable to recognize.

### Caveats.

In the present study, we encountered a number of issues that confounded data analysis and were challenging to circumvent. Technical artifacts in the 16S rRNA data set were obvious and persistent (Fig. 4C); future studies should aim to sequence all samples on a single Illumina run, in order to avoid the introduction of technical artifacts that may confound data analysis. Low-diversity microbial communities were unexpectedly recovered from “control” samples, requiring stringent data filtering to remove all potential contaminant OTUs. The collection protocol for ambient air control samples may have inadvertently collected species from airborne dust; alternatively, the manufactured swab samples we utilized may not have been entirely sterile. Regardless, our stringent data filtering protocol ultimately resulted in a significant reduction of sequences per sample—representing a level of coverage that was sufficient for microbial ecology analyses but a sequencing depth that was far from ideal (particularly for the 16S rRNA amplicons which were not sequenced as exhaustively as those of the eukaryotic 18S rRNA). Thus, our analyses may have failed to detect some microbial diversity and community patterns that might only have become apparent with extremely deep sequencing of ATM keypads (e.g., rare biosphere biomarker taxa), especially in lower-coverage bacterial/archeal samples.

### Future work.

The present study aimed to solely characterize microbial diversity (living or dead) on ATM keypads; we had no way of measuring levels of active versus dormant microbes or of assessing what could be considered “residual” or “transitory” species aside from obvious food species (e.g., microbes/fungi transported through air/dust). Additional, complementary analyses will be needed to determine whether the detected species are metabolically active and whether some species can survive on ATM keypads for extended periods of time. Although we detected DNA signatures from potential pathogens on ATM keypads, more-targeted studies will be needed to confirm the source, viability, and distribution of such pathogenic species on urban surfaces. For example, the human pathogen *Trichomonas vaginalis* cannot be differentiated from closely related zoonotic species using 18S rRNA loci alone (Maritz et al., submitted) and in this study we could not confirm the likely source of eukaryotic pathogen species. Future studies of the urban microbiome should also include an expanded examination of ATMs in both indoor and outdoor locations. LEfSe biomarkers in our 16S rRNA data set (see Fig. S5 in the supplemental material), as well as previous studies of urban transit systems (28), have indicated that indoor/outdoor location is a significant factor structuring microbial communities. Depletion/enrichment of indoor and outdoor biomarkers may be especially relevant for eukaryotic taxa, especially for pollen and airborne fungal spores. Unfortunately, we were unable to assess such patterns in the present study, as the subset of eukaryotic 18S rRNA data included only samples from indoor ATMs. In addition, there is a need for expanded studies that investigate shifts in ATM microbial communities over time, as well as for more-intense sampling covering a wider geographic area (including sample collection across other cities worldwide). Our study provided only a small snapshot of microbial communities, in terms of time points and the number of ATMs sampled. Nonetheless, the present data set has provided a significant baseline for microbial diversity on ATM keypads, broadly encompassing two taxonomic domains. The detection of microbial biomarker taxa in this study provides a jumping-off point for future study design and hypothesis testing, hinting at potential large-scale trends that may influence the distribution and persistence of microbes on highly trafficked urban surfaces.

## MATERIALS AND METHODS

### Sampling and metadata collection.

Microbial swab samples were collected during June and July 2014 at automated teller machines (ATMs) in eight neighborhood tabulation areas (NTAs) of New York City, representing three boroughs (Manhattan, Queens, and Brooklyn; Fig. 1). NTAs are geographic units designated by the Department of City Planning that are used to project population size and demographics at the small-area level (http://www1.nyc.gov/site/planning/data-maps/open-data/dwn-nynta.page). NTAs are units with a minimum population size of 15,000 and are used to collect and collate census/survey data; they do not necessarily reflect historical boundaries of NYC neighborhoods. In this study, sampling locations were preselected based on known neighborhood demographics, and ATM keypads were sampled randomly within areas proximate to a subway station within each NTA.

ATMs were sampled using sterile cotton swabs individually packaged in pairs (Covidien cotton-tipped applicators; Fisher Scientific catalog no. 22-037-924) and premoistened with 0.15 M NaCl solution–0.1% Tween 20. Each ATM keypad was sampled using 2 cotton swabs at a time, scrubbing all keys for a total of 10 s. A total of six control swabs were collected across different NTAs. For each control sample, a cotton swab was removed from sterile packaging, dipped in buffer solution, and held in ambient air for 10 s.

At each sampling location, the following metadata were also recorded: date, time, neighborhood (NTA), NYC borough, type of ATM keypad (metal or plastic), ATM location (indoor or outdoor), and site type (hospital, bank, convenience store, etc.). Global Positioning System (GPS) coordinates were not collected during sampling, in order to anonymize the location of all ATMs used in this study.

In order to analyze microbial community patterns in conjunction with NYC neighborhood demographics, additional metadata about each sampling area were collated from online sources. Demographic information for each NTA was obtained from the New York City Open Data portal (Fig. 1; public data set: https://data.cityofnewyork.us/City-Government/Demographics-and-profiles-at-the-Neighborhood-Tabu/hyuz-tij8), where all population information is derived from 5-year estimates (2008 to 2012) from the United States Census Bureau’s American Community Survey (https://www.census.gov/programs-surveys/acs/). For this study, we selected a subset of metadata that was potentially relevant to the assessment of microbial community patterns on ATM keypads, such as population density, and indicators of genetic factors, lifestyle, or habits that may be influenced by cultural or age demographics of the population within each NTA. The following broad population statistics were obtained for each NTA: population recorded during the 2000 United States Census; population recorded during the 2010 United States Census; and population change (both number and percent change) between the 2000 United States Census and the 2010 United States Census. An additional set of population demographics related to race, ancestry, and age was also obtained for each NTA as follows: percentage of the population who reported belonging to a single race; top three race groups comprising the largest numbers and percentages of the population; top three age groups comprising the largest percentages of the population (quantified by age group and percentage); percentage of foreign-born residents; geographic origin of the primary foreign-born group (quantified by race); and predominant ancestry group (self-reported ethnic origin recorded for a small subset of the population).

### DNA extraction, PCR amplification, and sequencing.

We extracted DNA using a MoBio PowerLyzer PowerSoil extraction kit (catalog no. 12855-100) according to manufacturer instructions. One cotton swab from each sample site was clipped and placed into an individual well of a 96-well bead plate for DNA extractions (the second swab from each sample site was retained in frozen storage for future studies). For each ATM sample, the same environmental DNA extraction method was used to generate PCR amplicons for both the 16S rRNA and 18S rRNA genes.

The V4 fragment of the 16S rRNA gene was amplified using the 515F/806R primer set (54). All primers and protocols used for amplification and sequencing represent standardized workflows obtained from the Earth Microbiome Project (EMP) website (http://www.earthmicrobiome.org/emp-standard-protocols/16s/). Amplification was done in triplicate following the EMP protocol. After amplification, reactions were quantified using a Pico Green (Invitrogen) assay kit, equal amounts of amplicons were pooled, and the final pool was cleaned using a QIAquick PCR purification kit (Qiagen). Amplicons were sequenced using a paired-end Illumina MiSeq platform, as previously described (54).

The V9 fragment of the 18S rRNA gene was amplified using Illumina primer constructs containing the universal primers 1391f-EukBr. Library synthesis and amplification using 2 µl of input DNA were done in triplicate following the EMP protocol, available online (http://www.earthmicrobiome.org/emp-standard-protocols/18s/). After amplification, triplicate PCRs were pooled prior to purification and quantification and prepared for sequencing following the protocol described by Maritz et al. (submitted for publication). Purified libraries that showed high proportions of adapter dimers were size selected using a 2% agarose dye-free gel on a BluePippin instrument (Sage Science).

All barcoded rRNA libraries were subsequently sequenced on an Illumina MiSeq platform (2-by-150 paired-end reads, with a 5 to 10% PhiX spike-in, based on the strategy outlined in reference 54). The 16S rRNA samples were sequenced on two separate MiSeq runs (as they represented two distinct sampling time points), while all 18S rRNA samples were pooled and sequenced together on a single MiSeq run (2-by-100 paired-end reads with a 6% PhiX spike-in).

### Data filtering and processing.

For both the 16S rRNA and 18S rRNA datasets, the majority of data filtering and processing steps were carried out using QIIME v1.8 (55). To process raw Illumina data, paired-end reads were merged using join_paired_ends.py, a minimum overlap of 10 bp, and a 15% error rate in the overlapping bases. Joined reads were subsequently demultiplexed using split_libraries_fastq.py with the rev_comp_mapping_barcodes flag; a minimum Phred quality score of 20, allowing 5 poor-quality bases before read truncation; and an 0.70 minimum fraction of consecutive high-quality base calls to include reads. Merged sequences that passed quality filtering thresholds were subsequently clustered into operational taxonomic units (OTUs) using the pick_open_reference_otus.py workflow (56) with 10% subsampling and *de novo* clustering of failure reads. Any resulting singleton OTUs were discarded (the minimum cluster size was set at 2 reads). A 97% clustering cutoff was used for 16S rRNA OTU picking, representing a standard approach in microbial ecology studies of bacteria/archaea. A more stringent 99% clustering cutoff was used for 18S rRNA OTU picking, as the 18S rRNA gene is typically more conserved and less variable in eukaryotic genomes (57) and because the use of a higher clustering cutoff is also in line with many comparable environmental sequencing studies of eukaryotes (58). For OTU picking, initial reference-based OTU clustering was carried out against Greengenes 13_8 (97% OTUs) for 16S rRNA data (59) and SILVA 119 (99% OTUs) for 18S rRNA data (60). Summaries of OTUs and quality-processed reads obtained across samples are provided in Fig. S6  (16S rRNA) and Fig. S7 (18S rRNA) in the supplemental material, and a more detailed record of demultiplexing and processing is provided in Table S1 in the supplemental material.

10.1128/mSphere.00226-16.1 Table S1  Summary of sequence reads and OTU counts for all ATM swab samples. Summarized data reflect the outputs of open reference OTU picking in QIIME 1.8 for 16S rRNA (bacterial/archaeal) and 18S rRNA (eukaryotic) amplicons. Summaries are also reported for sequential downstream filtering steps to remove alignment failures and chimeras, followed by abundance filtering and removal of all OTUs present in blank control samples. Download Table S1, PDF file, 0.1 MB. Copyright © 2016 Bik et al. 2016 Bik et al. This content is distributed under the terms of the Creative Commons Attribution 4.0 International license.

10.1128/mSphere.00226-16.7 Figure S6  Sequence reads and OTU counts for 16S rRNA data. Reads and OTUs were calculated following open reference OTU clustering in the QIIME pipeline, carried out using uclust with a 97% similarity cutoff. OTU counts exclude clusters containing <2 reads. Yellow circles indicate blank control samples. Data shown represents the raw results from open reference OTU picking (see the first four columns of Table S1), without application of any downstream data filtering based on alignment failures, chimeras, or OTU abundances. Download Figure S6, PDF file, 0.2 MB. Copyright © 2016 Bik et al. 2016 Bik et al. This content is distributed under the terms of the Creative Commons Attribution 4.0 International license.

10.1128/mSphere.00226-16.8 Figure S7  Sequence reads and OTU counts for 18S rRNA data. Reads and OTUs were calculated following open reference OTU clustering in the QIIME pipeline, carried out using uclust with a 99% similarity cutoff. OTU counts exclude clusters containing <2 reads. Yellow circles indicate blank control samples. Data shown represent the raw results from open reference OTU picking (see the first four columns of Table S1), without application of any downstream data filtering based on alignment failures, chimeras, or OTU abundances. Download Figure S7, PDF file, 0.2 MB. Copyright © 2016 Bik et al. 2016 Bik et al. This content is distributed under the terms of the Creative Commons Attribution 4.0 International license.

For 16S rRNA OTUs, taxonomy was assigned to representative sequences using QIIME’s uclust consensus taxonomy assigner and Greengenes rRNA database version 13_8 (97% OTU representative sequences). For 18S rRNA OTUs, taxonomy was assigned in two steps (based on methods described in reference 61). First, BLAST was used to compare OTU representative sequences to a manually curated version of the SILVA 111 database, containing only eukaryotic sequences. In this curated database, taxonomic hierarchies were standardized and corrected, and some protist sequences were manually added to improve representation of some groups (Maritz et al., submitted). The taxonomy from the top BLAST hit was taken for any OTUs matching the curated database. Second, any OTUs without a BLAST hit were subsequently compared to the entire SILVA 111 database (99% OTU representative sequences) containing reference sequences from all three domains. The taxonomy from the top BLAST hit was again accepted for any OTUs matching the reference database, and failed sequences were denoted as “unassigned.” In both taxonomy assignment steps, the minimum E value cutoff for BLAST searches was set at 1e-20, and the top BLAST hit was taken for each representative OTU sequence; sequences were denoted as “unassigned” if they did not return any results meeting this E value cutoff criterion.

Representative OTU sequences from all ATM samples were subsequently aligned using the PYNAST aligner (62) and the same reference databases used for OTU picking. Chimera checking was also carried out using ChimeraSlayer (63) for 16S rRNA and 18S rRNA data (a database-dependent method) and additionally using the Blast Fragments method in QIIME for 18S rRNA data (a database-independent chimera checking method, given the sparse nature of 18S rRNA databases and the less variable nature of eukaryotic rRNA genes).

Before any microbial community diversity analyses and comparisons were carried out, the initial OTU tables resulting from open-reference OTU picking were filtered to remove unwanted and poor-quality sequences. First, nontarget OTUs were filtered from each rRNA data set on the basis of taxonomic assignments. Chloroplast, mitochondrial, and “Unassigned” sequences were removed from the 16S rRNA data set. Bacterial, archaeal, and *Archaeplastida* sequences were removed from the 18S rRNA data set. Next, any OTUs that failed to align to reference rRNA databases or were flagged as chimeric were removed. OTU tables were subsequently subjected to abundance-based filtering, removing low-abundance OTUs representing less than 0.0005% of total reads in the data set (using the min_count_fraction 0.000005 flag in QIIME for filter_otus_from_otu_table.py, following the methods described in reference 64). Finally, initial exploration of data and SourceTracker analysis (see below) suggested that a large proportion of environmental OTUs were derived from blank samples sequenced as controls. For maximal stringency, we additionally removed all OTUs that appeared in control samples, resulting in significant data reduction for both 16S and 18S rRNA (Table S1).

### Microbial community analyses.

For representative sequences of OTUs that satisfied all filtering criteria, phylogenetic trees were constructed from gap-filtered alignments using the FastTree algorithm (65) with default parameters. Alpha diversity metrics, including rarefaction curves and taxonomy summaries, were calculated using the alpha_rarefaction.py script in QIIME. Beta diversity analyses, including weighted and unweighted Unifrac principal-coordinate analyses, were carried out using the beta_diversity_through_plots.py workflow script in QIIME. For both alpha and beta diversity analyses, OTU tables were rarefied on the basis of the sample with the lowest number of total sequence reads (the minimum rarefaction levels were 1,700 sequences for 16S rRNA data and 8,900 sequences for 18S rRNA data). Distance matrices from Unifrac PCoAs were imported into R studio and visualized using the phyloseq (66) and ggplot2 packages. To further test for dissimilarity between sample groups in unweighted Unifrac beta diversity analyses, we carried out nonparametric PERMANOVA tests (using compare_categories.py in QIIME) and assessed intragroup and intergroup distances using nonparametric *t* tests (make_distance_boxplots.py in QIIME).

The 16S rRNA data set was additionally subjected to SourceTracker analysis (67), in order to identify the potential origin of microbial communities on ATM keypads. OTUs obtained from this study were compared to 12 public datasets compiled for a previous meta-analysis study (68). The chosen published datasets represent a range of potential source environments, including the human body (44, 45, 69, 70), surfaces in the built environment (10, 15, 16, 53, 71), soils (72), and indoor/outdoor air samples (13, 73). Demultiplexed 16S rRNA sequences from ATM keypads were subjected to closed-reference OTU picking (using the pickTheseOTUs.sh script as described in reference 68), and the resulting OTU table was merged with closed-reference tables from published studies (downloaded from GitHub: https://github.com/jfmeadow/BEMAFinalAnalysis/tree/master/individual_biom_tables). Closed-reference OTU picking was necessary for SourceTracker analysis, as this database-dependent approach avoids potential erroneous/chimeric OTUs and further enables the concurrent analysis of datasets obtained using different 16S rRNA primer sets, read lengths, and sequencing platforms. Among the ATM samples in this study, blank controls were also marked as potential sources of microbial communities in order to identify any potential contamination introduced during PCR and sequencing. Unfortunately, SourceTracker analysis was not possible for our eukaryotic data set because of the comparative lack of published 18S rRNA studies from urban and built environments, database limitations, and the prevalence of other markers used for other eukaryotic studies (internal transcribed spacer [ITS] rRNA, etc.).

Linear discriminant analysis (LDA) effect size (LEfSe) analysis (74) was carried out on both 16S and 18S rRNA datasets, to assess whether any microbial biomarkers could be identified across sample categories. LEfSe analyses were carried out using an online Galaxy server (http://huttenhower.sph.harvard.edu/galaxy/). Per-sample normalization was carried out on all input OTU tables, and LDA effect size was calculated using the strict “all-against-all” strategy for multiclass analysis and default parameters and thresholds.

### Accession number(s).

Raw Illumina sequence data for both 16S and 18S rRNA genes have been deposited in the NCBI SRA under BioProject PRJNA330663 (SRA accession no. SRP079707). Additional documentation of all QIIME workflows, parameters, and OTU picking outputs has been compiled and deposited in FigShare (10.6084/m9.figshare.3498206).
