# Supplementary figures and images for: Microbial Community Patterns Associated with Automated Teller Machine Keypads in New York City
Source: mSphere. 2016 Nov 16;1(6):e00226-16. doi: 10.1128/mSphere.00226-16 (PMC5112336; doi:10.1128/mSphere.00226-16)

Figure S1

A

16S rRNA – Unweighted Unifrac

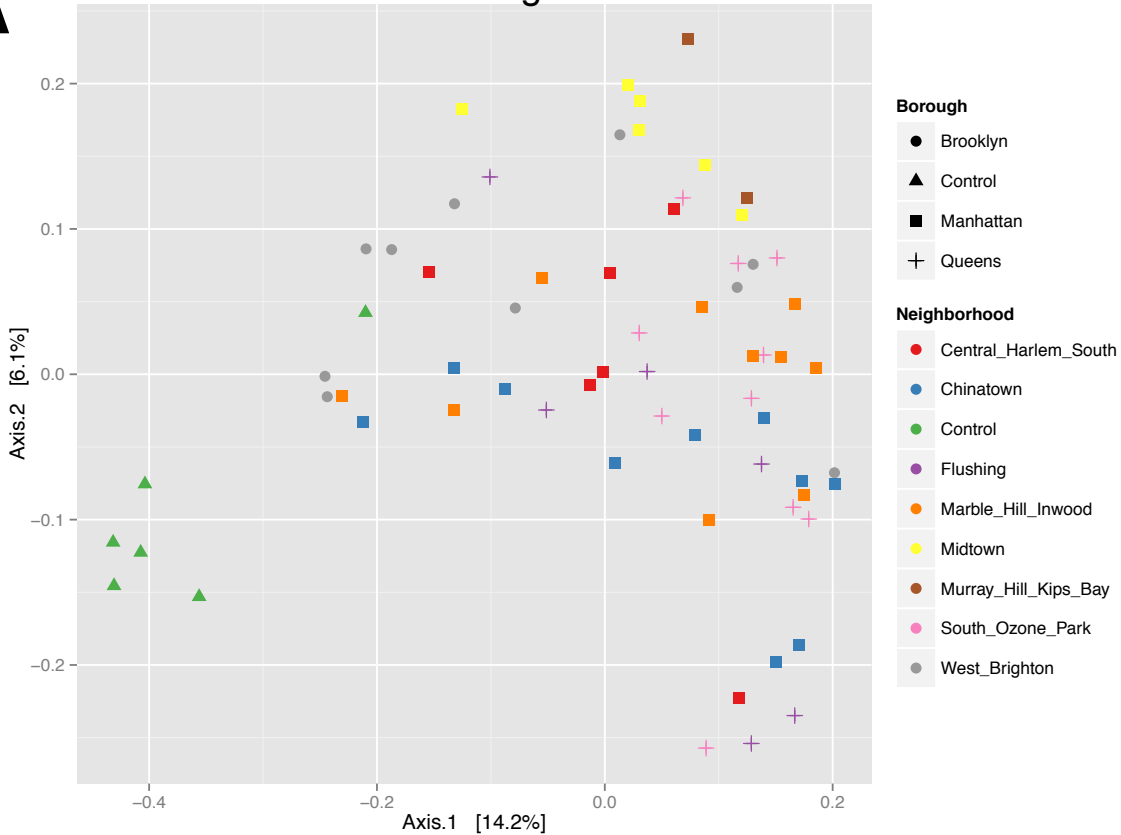

B

18S rRNA – Unweighted Unifrac

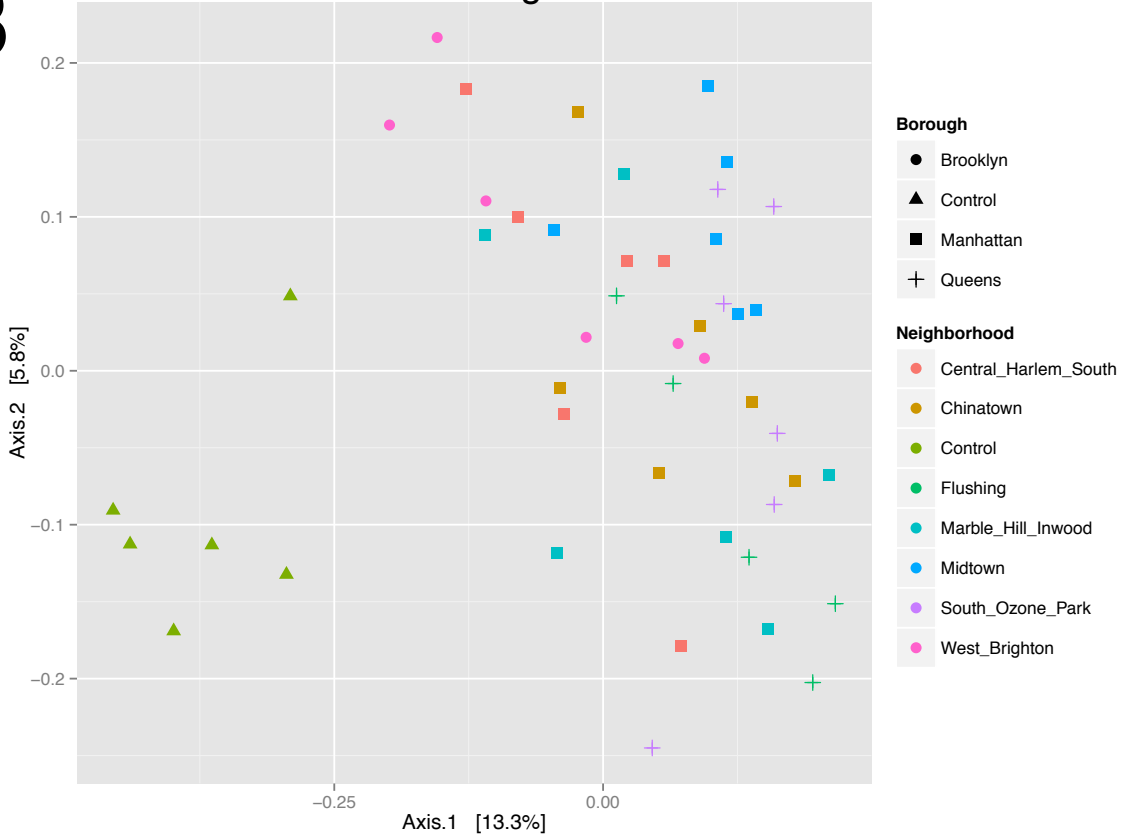

Supplement: Figure S1 [file sph001172182sf2.pdf]

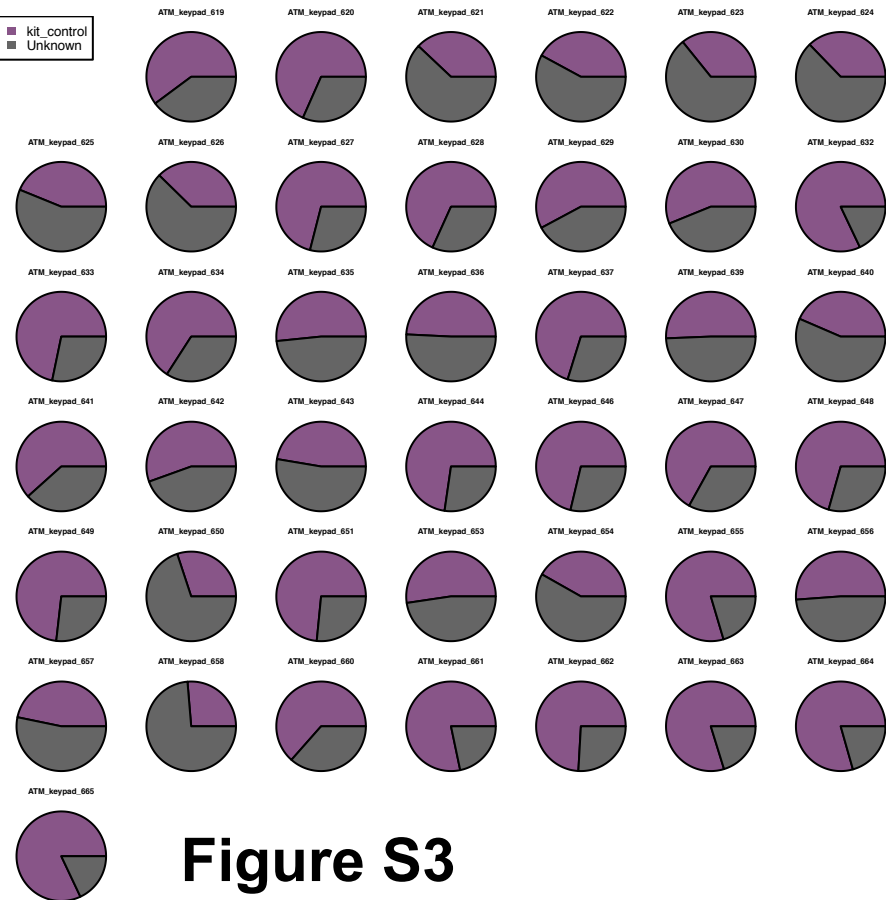

Supplement: Figure S3 [file sph001172182sf4.pdf]

Figure S4

A

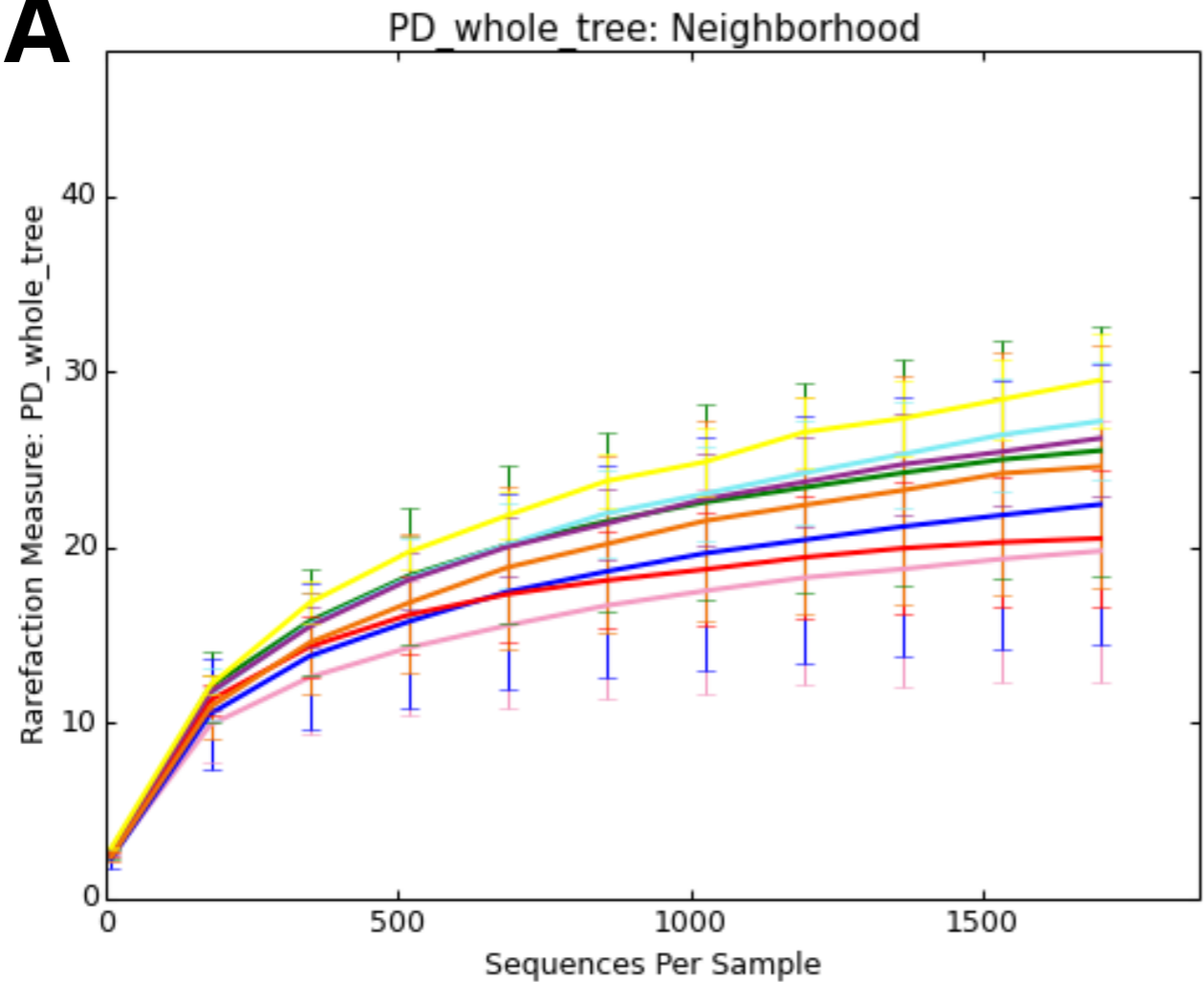

16S rRNA  
Legend

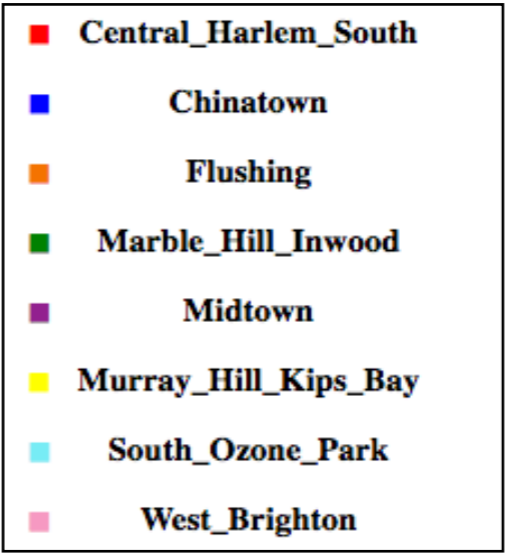

B

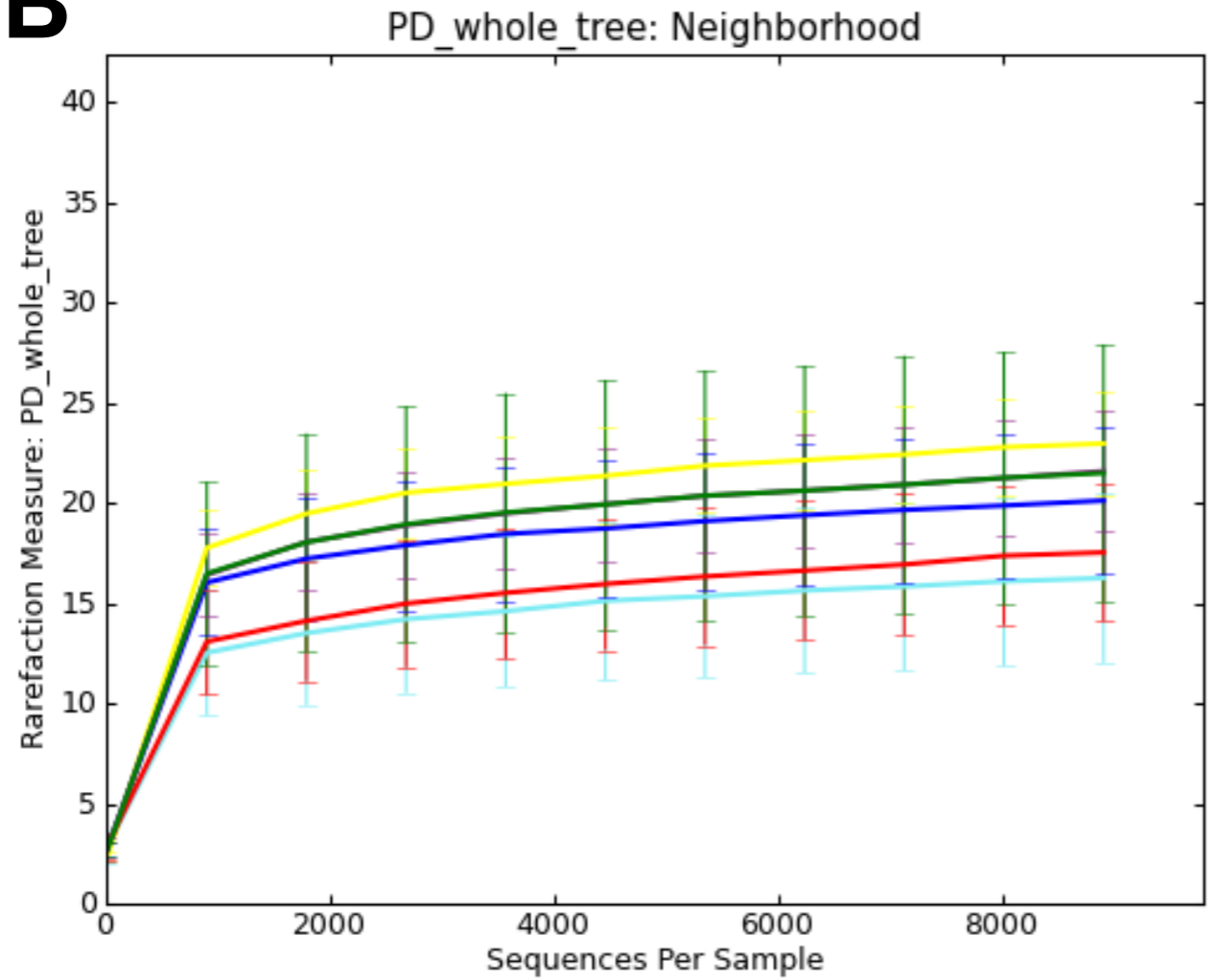

18S rRNA  
Legend

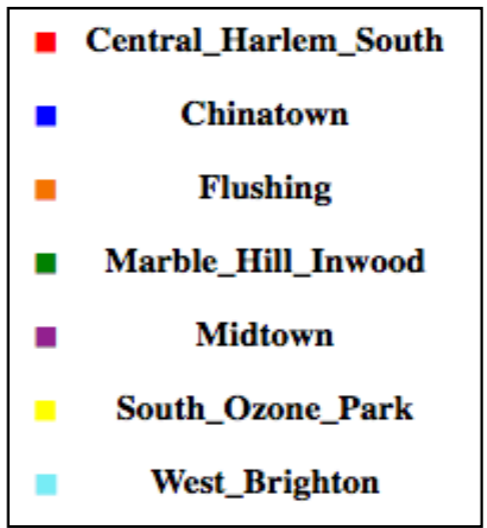

Supplement: Figure S4 [file sph001172182sf5.pdf]

Indoor Outdoor

Figure S5

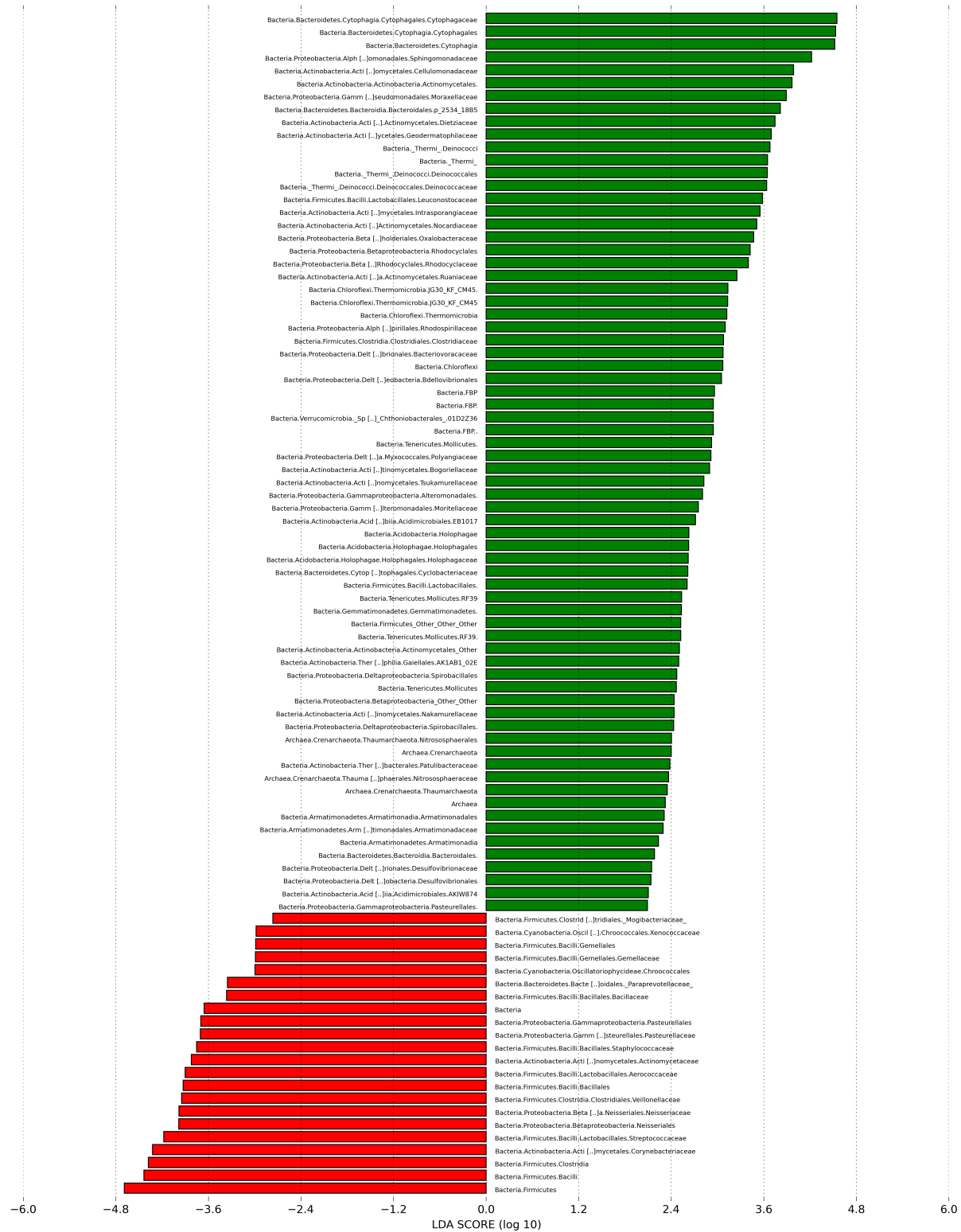

Supplement: Figure S5 [file sph001172182sf6.pdf]

**Figure S6**

# 16S rRNA - Reads/OTUs per sample

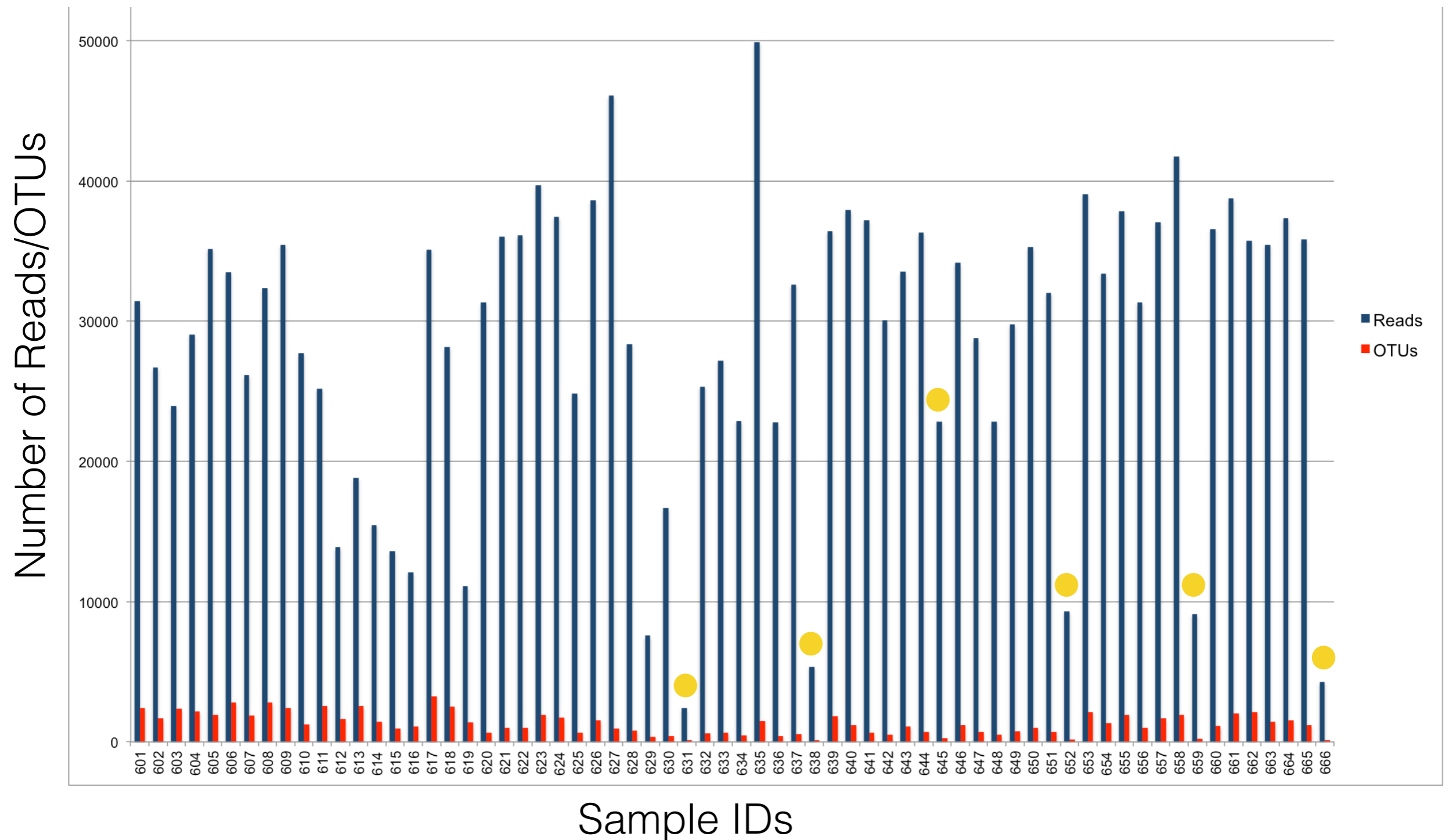

Supplement: Figure S6 [file sph001172182sf7.pdf]

**Figure S7**

# 18S rRNA - Reads/OTUs per sample

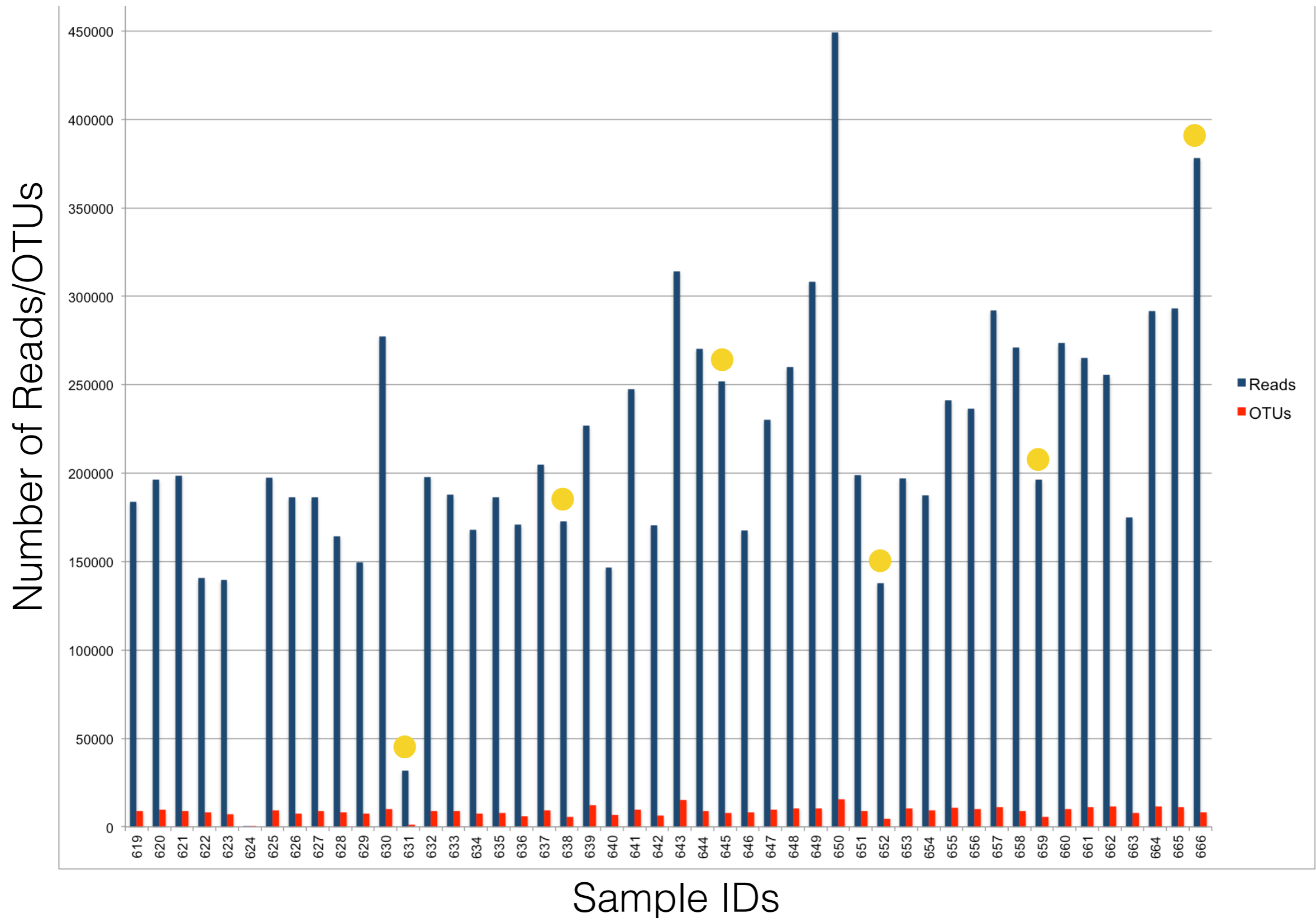

Supplement: Figure S7 [file sph001172182sf8.pdf]
