# Supplementary material for: Microbial Community Patterns Associated with Automated Teller Machine Keypads in New York City
Source: mSphere. 2016 Nov 16;1(6):e00226-16. doi: 10.1128/mSphere.00226-16 (PMC5112336; doi:10.1128/mSphere.00226-16)
Supplement: Table S1 [file sph001172182st1.pdf]

		Open Reference OTU picking				Alignment Failures and Chimeras Removed				Kit Control OTUs Removed + Abundance Filtering (<0.0005%)			
Sample ID	Neighborhood	16S Reads	16S OTUs (97%)	18S Reads	18S OTUs (99%)	16S Reads	16S OTUs (97%)	18S Reads	18S OTUs (99%)	16S Reads	16S OTUs (97%)	18S Reads	18S OTUs (99%)
601	Murray_Hill_Kips_Bay	31427	2402	*no_data*	*no_data*	27484	1818	*no_data*	*no_data*	11228	1189	*no_data*	*no_data*
602	Murray_Hill_Kips_Bay	26672	1660	*no_data*	*no_data*	24436	1230	*no_data*	*no_data*	9206	799	*no_data*	*no_data*
603	Chinatown	23952	2388	*no_data*	*no_data*	18514	1837	*no_data*	*no_data*	8763	1187	*no_data*	*no_data*
604	Chinatown	29053	2196	*no_data*	*no_data*	25343	1643	*no_data*	*no_data*	12928	1097	*no_data*	*no_data*
605	Chinatown	35163	1939	*no_data*	*no_data*	31534	1414	*no_data*	*no_data*	12498	898	*no_data*	*no_data*
606	Chinatown	33478	2816	*no_data*	*no_data*	24561	1975	*no_data*	*no_data*	12062	1240	*no_data*	*no_data*
607	Marble_Hill_Inwood	26171	1857	*no_data*	*no_data*	22303	1435	*no_data*	*no_data*	10104	930	*no_data*	*no_data*
608	Marble_Hill_Inwood	32360	2817	*no_data*	*no_data*	28107	1982	*no_data*	*no_data*	11453	1285	*no_data*	*no_data*
609	Marble_Hill_Inwood	35414	2422	*no_data*	*no_data*	31402	1810	*no_data*	*no_data*	13726	1191	*no_data*	*no_data*
610	Marble_Hill_Inwood	27700	1262	*no_data*	*no_data*	24927	1048	*no_data*	*no_data*	11256	680	*no_data*	*no_data*
611	South_Ozone_Park	25193	2553	*no_data*	*no_data*	20527	1719	*no_data*	*no_data*	8744	1156	*no_data*	*no_data*
612	South_Ozone_Park	13874	1644	*no_data*	*no_data*	12472	1229	*no_data*	*no_data*	5293	767	*no_data*	*no_data*
613	South_Ozone_Park	18822	2539	*no_data*	*no_data*	14860	1792	*no_data*	*no_data*	7167	1206	*no_data*	*no_data*
614	South_Ozone_Park	15476	1436	*no_data*	*no_data*	14229	1147	*no_data*	*no_data*	5778	746	*no_data*	*no_data*
615	West_Brighton	13609	958	*no_data*	*no_data*	12471	760	*no_data*	*no_data*	4933	485	*no_data*	*no_data*
616	West_Brighton	12099	1089	*no_data*	*no_data*	9051	844	*no_data*	*no_data*	4152	547	*no_data*	*no_data*
617	West_Brighton	35105	3241	*no_data*	*no_data*	29117	2366	*no_data*	*no_data*	14675	1488	*no_data*	*no_data*
618	West_Brighton	28147	2517	*no_data*	*no_data*	24819	1795	*no_data*	*no_data*	9822	1209	*no_data*	*no_data*
619	Flushing	11092	1409	183774	8918	9389	1047	132459	5604	5421	712	23301	872
620	Flushing	31351	667	196362	9649	26719	516	140357	6465	9281	303	20037	530
621	Flushing	36021	1018	198581	8990	30429	755	83111	4045	21977	480	35753	527
622	Flushing	36119	979	140593	8266	27027	728	89521	4913	10636	460	26103	742
623	Flushing	39699	1936	139551	7138	32270	1564	110997	4898	16056	1077	22040	883
624	Flushing	37430	1754	99	54	31027	1362	72	39	16652	912	26	18
625	Chinatown	24818	667	197418	9292	20799	547	162916	7347	11554	341	34490	873
626	Chinatown	38588	1545	186368	7495	28626	1184	160337	5678	11731	796	35126	871
627	Chinatown	46072	934	186395	9042	44769	660	144513	6987	41363	399	21333	834
628	Chinatown	28370	791	164433	8270	22598	642	130897	6082	9936	415	21971	490
629	Chinatown	7575	371	149650	7570	6558	325	112284	5356	3070	191	28942	620
630	Chinatown	16689	415	277394	10246	11372	337	229592	8167	5491	204	26205	605
631	Control	2428	115	31835	1150	2272	96	26739	822	0	0	0	0
632	West_Brighton	25335	592	197913	8800	22902	436	155345	6708	7262	250	10385	525
633	West_Brighton	27201	677	187704	9079	24634	531	140404	6738	8919	318	20122	666
634	West_Brighton	22877	455	167832	7448	18909	339	117454	5141	7143	185	18163	313
635	West_Brighton	49879	1493	186237	7677	47330	988	133372	5667	9037	571	45105	627
636	West_Brighton	22778	415	170764	6045	21207	341	115910	3658	8654	181	29304	383
637	West_Brighton	32600	536	204935	9477	30364	429	152902	7054	4912	252	19865	635
638	Control	5368	130	172812	5518	5166	89	146123	4404	0	0	0	0
639	Marble_Hill_Inwood	36418	1811	226884	12332	26827	1432	180668	9140	12225	1002	34587	1259
640	Marble_Hill_Inwood	37904	1190	146701	6683	29898	898	135247	5557	11469	598	25293	815
641	Marble_Hill_Inwood	37191	652	247489	9588	27379	511	185828	7356	14239	328	19210	499
642	Marble_Hill_Inwood	30074	490	170511	6389	28055	372	148126	5252	14958	210	41590	421
643	Marble_Hill_Inwood	33512	1102	314253	15381	26007	880	231063	10541	13010	591	37029	1138
644	Marble_Hill_Inwood	36311	716	270407	8961	34539	499	175644	4961	1740	250	8948	464
645	Control	22852	260	252057	7711	21833	196	140028	4228	0	0	0	0
646	Central_Harlem_South	34153	1199	167476	8162	30815	930	136880	6595	13393	607	13475	743
647	Central_Harlem_South	28771	726	230253	9814	24974	595	141878	5821	8220	367	17565	462
648	Central_Harlem_South	22840	487	259968	10513	20287	389	229708	9008	7368	211	29236	631
649	Central_Harlem_South	29765	737	308384	10331	27425	608	281769	8838	12048	401	18818	654
650	Central_Harlem_South	35265	1006	449150	15601	25675	794	288272	9730	14792	549	53667	1136
651	Central_Harlem_South	32013	703	199071	8909	27710	521	158816	6863	7019	305	14861	617
652	Control	9295	176	137799	4436	9091	135	91595	2928	0	0	0	0
653	South_Ozone_Park	39067	2144	196975	10303	30769	1565	164652	8065	13420	1057	29326	1043
654	South_Ozone_Park	33392	1341	187464	9162	21982	984	106171	5319	8829	656	20153	716
655	South_Ozone_Park	37852	1922	241129	10684	35787	1400	207752	8629	14005	908	15429	848
656	South_Ozone_Park	31347	1009	236624	10161	25113	785	169729	7475	13287	518	39302	809
657	South_Ozone_Park	37063	1699	292081	11264	32917	1162	226361	8469	19303	765	37748	867
658	South_Ozone_Park	41727	1913	271228	8887	28857	1362	220430	6054	19689	891	92905	722
659	Control	9117	195	196170	5517	8450	158	148440	4114	0	0	0	0
660	Midtown	36575	1157	273480	10122	34580	818	260831	9028	5246	526	26497	519
661	Midtown	38765	2039	265147	11210	35394	1438	214236	8911	9329	921	19184	829
662	Midtown	35746	2100	255515	11597	32838	1594	223378	9262	13729	1060	26149	887
663	Midtown	35445	1456	175120	7951	33678	1102	164828	6989	11614	747	9087	642
664	Midtown	37334	1519	291615	11604	34979	1133	263500	9818	10690	783	18874	795
665	Midtown	35841	1176	293288	11132	29155	857	267869	9422	9620	566	15880	605
666	Control	4270	110	378315	8321	3638	76	256733	5896	0	0	0	0
