# Supplementary material for: Microbial Community Patterns Associated with Automated Teller Machine Keypads in New York City
Source: mSphere. 2016 Nov 16;1(6):e00226-16. doi: 10.1128/mSphere.00226-16 (PMC5112336; doi:10.1128/mSphere.00226-16)
Supplement: Figure S2 [file sph001172182sf3.pdf]

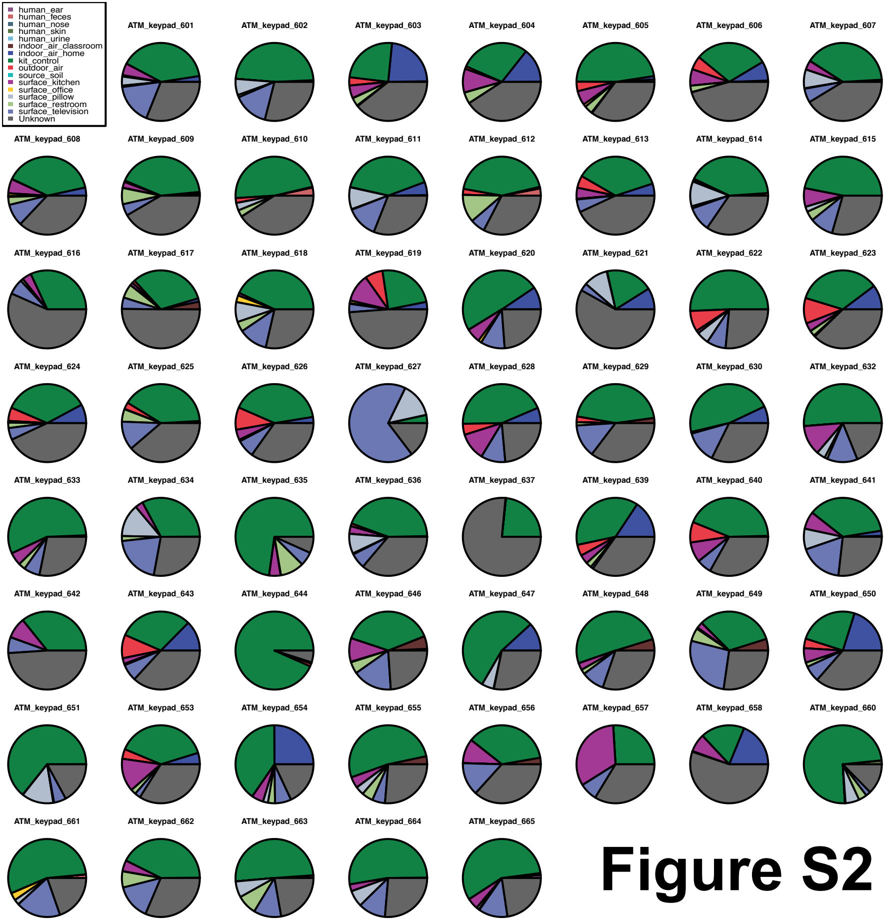
human_ear
human_feces
human_nose
human_skin
human_urine
indoor_air_classroom
indoor_air_home
kit_control
outdoor_air
source_soil
surface_kitchen
surface_office
surface_pillow
surface_restroom
surface_television
Unknown
ATM_keypad_601
ATM_keypad_602
ATM_keypad_603
ATM_keypad_604
ATM_keypad_605
ATM_keypad_606
ATM_keypad_607
ATM_keypad_608
ATM_keypad_609
ATM_keypad_610
ATM_keypad_611
ATM_keypad_612
ATM_keypad_613
ATM_keypad_614
ATM_keypad_615
ATM_keypad_616
ATM_keypad_617
ATM_keypad_618
ATM_keypad_619
ATM_keypad_620
ATM_keypad_621
ATM_keypad_622
ATM_keypad_623
ATM_keypad_624
ATM_keypad_625
ATM_keypad_626
ATM_keypad_627
ATM_keypad_628
ATM_keypad_629
ATM_keypad_630
ATM_keypad_632
ATM_keypad_633
ATM_keypad_634
ATM_keypad_635
ATM_keypad_636
ATM_keypad_637
ATM_keypad_639
ATM_keypad_640
ATM_keypad_641
ATM_keypad_642
ATM_keypad_643
ATM_keypad_644
ATM_keypad_646
ATM_keypad_647
ATM_keypad_648
ATM_keypad_649
ATM_keypad_650
ATM_keypad_651
ATM_keypad_653
ATM_keypad_654
ATM_keypad_655
ATM_keypad_656
ATM_keypad_657
ATM_keypad_658
ATM_keypad_660
ATM_keypad_661
ATM_keypad_662
ATM_keypad_663
ATM_keypad_664
ATM_keypad_665

Figure S2
